# Mortuary Statistics

**Published:** 1880-05

**Authors:** 


MORTUARY STATISTICS.—*Messrs. Editors*: I beg leave, through your public-spirited journal, to call the attention of the proper authorities to a few practical and vitally important suggestions, which, if adopted, may add much to the prosperity of our community.

In a large and rapidly increasing population like that of ours, it is almost impossible to overestimate the importance of correct and entirely reliable MORTUARY STATISTICS; and it becomes the duty of the medical profession, or at least such of its members as are disposed to keep pace with the progressive march of a humane and enlightened calling, to inform and instruct the masses in regard to such measures as may be best calculated to promote and preserve the general health.

Now, these reports, as published, may be, and probably are, strictly correct, so far as the *number* of interments is concerned, but they are very unsatisfactory when the *names* of the diseases are considered. Attention to this latter particular is highly important, in several points of view, and we will briefly mention a few reasons why a radical change should be made. It is not an unfrequent occurrence for the medical historian to examine the mortuary records of a city, with a view to drawing comparisons with other cities in regard to the prevalence of certain diseases, as well as for various other purposes of medical or general information. It is at once obvious that where the *names* of diseases are *improperly* or *incorrectly* reported by the sextons, no *reliable* data can be obtained from them as to the character of the disease of which the patient died. The remedy we propose is simple in its character, easy of application and certain in its results, and is as follows, viz: Make a law requiring every physician losing a case to give a written statement of the *name* of the disease, and as correctly as possible the *age* and *nativity* of the patient. Require the sexton to record such certificate in his books of interments, and by publishing the name of the attending physician in conjunction with the report of the case, the published accounts of interments would be rendered as reliable as a reference to the records themselves; and perhaps even more so, as the attending physician would then have an opportunity of seeing whether his certificate had been properly recorded by the sexton.

Again, make it obligatory on the sextons or keepers of the cemeteries, to inter no body without the certificate of a physician, as above, or the certificate of the coroner, where no physician was in attendance on the case. The foregoing suggestions would, if adopted, prevent the possibility of premature or other improper burials, and also furnish *reliable statistics* for *future use* or examination. It is hoped that the proper authorities will turn their attention to this subject, and thereby confer a benefit on the entire community in which we live.

Very respectfully, H. L. B.

The above article was published in the *Gazette* newspaper of this city about a decade ago, and though all its suggestions have not since been enacted into law, sufficient has been accomplished to give us tolerably correct returns of the fatal diseases taking place in the city each week. If we are correctly informed, some queer names of diseases still occur from time to time on the death certificate, a few of which would be amusing on a less grave subject. But why not publish the name of the physician, midwife, or coroner furnishing a certificate of a death? Perhaps the new names would then be better understood, and might in time find a place in our local mortuary nomenclature. But levity aside, it is due to the living and to future generations in this large and growing city, that our mortuary statistics should be absolutely correct, or at least as nearly so as is possible of attainment; and the only means of accomplishing this important end is to publish the names of all persons endorsing death certificates. There are potent reasons why that should be done, and we will endeavor to present some of them in a future issue in connection with the registration of marriages and births. In conducting medical journals attention should not only be given to matters immediately appertaining to the profession and coming under the purview of their editors at the present, but the probabilities that might be legitimately forecast of future events and occurrences should likewise claim and receive a proper share of attention and consideration at their hand. Such being our conception of the duties and responsibilities of our office, we shall endeavor to conduct our journal in such a manner as to make it a record not only of passing professional events, but also a chronicle of some of those of the approaching future. We feel conscious in inditing our editorials that many of them will, and some of them probably ought to, be read with interest even in the remote future. And such being the case we shall present facts as they may be known to us, or on the responsibility of others of reliability.

